# Current Marijuana Use and Alcohol Consumption Among Adults Following the Legalization of Nonmedical Retail Marijuana Sales — Colorado, 2015–2019

**DOI:** 10.15585/mmwr.mm7043a3

**Published:** 2021-10-29

**Authors:** Kacy A. Crawford, Jacqueline A. Gardner, Elisabeth A. Meyer, Katelyn E. Hall, Dahsan S. Gary, Marissa B. Esser

**Affiliations:** ^1^Colorado Department of Public Health and Environment; ^2^Division of Population Health, National Center for Chronic Disease Prevention and Health Promotion, CDC.

In Colorado, excessive alcohol use* contributed to $5 billion in economic costs in 2010 (*1*) and >1,800 deaths annually during 2011–2015 (*2*). The most common pattern of excessive drinking is binge drinking (consumption of four or more drinks on an occasion for women or five or more drinks for men) (*3*), which is associated with increased likelihood of using other substances, including marijuana (*4*). Retail (i.e., nonmedical) marijuana sales began in Colorado on January 1, 2014. The Colorado Department of Public Health and Environment (CDPHE) and CDC used data from Colorado’s 2015–2019 Behavioral Risk Factor Surveillance System (BRFSS) to examine current use of marijuana (including hashish) by drinking patterns among 45,991 persons aged ≥18 years who responded to questions about alcohol and marijuana use. The age-standardized, weighted prevalence of current marijuana use among persons who reported binge drinking (34.4%) was significantly higher than the prevalence among current non–binge drinkers (14.8%) and nondrinkers (9.9%). Evidence-based strategies recommended by the Community Preventive Services Task Force to reduce excessive alcohol use and tobacco use (e.g., increasing prices or reducing access) can reduce alcohol- and tobacco-related harms. Similar strategies might be effective in reducing marijuana use and its potential harms as well.

BRFSS is an annual state-based, random-digit–dialed landline and mobile telephone survey that collects information on health conditions and risk factors^†^ among the noninstitutionalized U.S. adult population aged ≥18 years. The current study was conducted using the following question, which was added to the Colorado survey: “During the past 30 days, on how many days did you use marijuana or hashish?” Current marijuana use was defined as having used marijuana, including hashish, on ≥1 day in the past 30 days. Frequency of marijuana use during the past 30 days^§^ was categorized as use on 1–3, 4–19, and ≥20 days (daily or near daily use). Questions from the BRFSS core questionnaire were used to measure alcohol consumption in the past 30 days, including the number of drinking days and the number of binge drinking episodes. Respondents were categorized into three groups by drinking pattern: 1) binge drinking^¶^ (consumption of four or more drinks for women or five or more drinks for men, on an occasion, one or more times in the past 30 days); 2) current drinking without binge drinking** (consumption of one or more alcoholic drinks on ≥1 day in the past 30 days but did not report binge drinking) (current non–binge drinking); and 3) nondrinking^††^ (no consumption of an alcoholic beverage in the past 30 days).

Colorado BRFSS data from 2015 to 2019 were combined, with an average response rate of 54% and a total sample of 56,513 respondents; 10,522 (19%) respondents were excluded because alcohol or marijuana data were missing or could not be analyzed (e.g., responses of “don’t know/not sure”), yielding a final sample of 45,991 respondents. CDPHE calculated age-standardized or age-specific, weighted percentages with 95% confidence intervals (CIs) to assess the prevalence of binge drinking by sociodemographic characteristics and of marijuana use by sociodemographic characteristics and drinking patterns. Age-standardized prevalence of marijuana use by drinking patterns was also assessed by cigarette smoking status.^§§^ Chi-square tests were used to assess significance of differences (p<0.05) in bivariate analyses. All analyses were performed using SAS (version 9.4; SAS Institute). This activity was reviewed by CDC and was conducted consistent with applicable federal law and CDC policy.^¶¶^

Overall, the age-standardized, weighted prevalence of binge drinking was 18.8% and of current marijuana use was 16.6% (Table). The prevalence of binge drinking was highest among adults aged 25–34 years (29.8%) and 18–24 years (25.8%), men (23.7%), non-Hispanic White adults (19.6%), and Hispanic adults (18.5%). The prevalence of current marijuana use was highest among young adults (aged 18–24 years) (28.0%) and men (20.2%), and lowest among Hispanic adults (13.3%). Approximately one third (34.4%) of adults who binge drank reported current marijuana use, which was significantly higher than that reported among current non–binge drinkers (14.8%) and nondrinkers (9.9%). Among adults who binge drank, current use of marijuana was most common among young adults aged 18–24 years (52.4%) and least common among adults aged ≥65 years (17.9%). By race/ethnicity, among persons who binge drank, current marijuana use was most common among non-Hispanic Black adults (44.4%) and least common among Hispanic adults (28.6%). Among adults who reported binge drinking and current marijuana use in the past 30 days, approximately one half (47.3%) reported daily or near daily (≥20 days) use of marijuana, 28.1% reported use on 4–19 days, and 24.6% reported use on 1–3 days.

**TABLE:** Weighted prevalence* of binge drinking and current marijuana use^†^ by drinking pattern,^§^ overall and by sociodemographic characteristics^¶^ — Colorado Behavioral Risk Factor Surveillance System, 2015–2019

Characteristic	Weighted %** (95% CI)				
	All respondents (N = 45,991)		Current marijuana use by drinking pattern		
	Binge drinking	Current marijuana use	Nondrinking	Current non–binge drinking	Binge drinking
	(n = 6,023)	(n = 5,586)	(n = 1,376)	(n = 2,400)	(n = 1,810)
**All**					
Crude^††^	17.6 (17.1–18.1)	15.8 (15.4–16.3)	9.5 (8.9–10.1)	14.2 (13.5–14.9)	33.9 (32.3–35.4)
Age-standardized	18.8 (18.2–19.3)	16.6 (16.0–17.1)	9.9 (9.2–10.5)	14.8 (14.1–15.5)	34.4 (32.8–36.0)
**Age group, yrs** ^††^					
18–24	25.8 (23.6–27.9)	28.0 (25.9–30.2)	12.2 (10.0–14.5)	31.1 (27.0–35.3)	52.4 (47.7–57.2)
25–34	29.8 (28.2–31.4)	24.4 (22.9–26.0)	16.0 (13.6–18.4)	20.9 (18.6–23.2)	37.8 (34.7–40.9)
35–44	22.1 (20.7–23.4)	16.8 (15.6–18.0)	9.8 (8.2–11.3)	15.8 (14.0–17.5)	30.2 (27.0–33.3)
45–54	17.2 (16.2–18.3)	12.1 (11.2–13.0)	10.1 (8.7–11.5)	9.7 (8.5–10.9)	23.0 (20.1–25.9)
55–64	11.2 (10.4–12.0)	12.9 (12.1–13.7)	9.3 (8.2–10.5)	12.7 (11.5–13.8)	26.8 (23.5–30.0)
≥65	4.3 (3.9–4.7)	6.4 (5.9–6.9)	4.2 (3.6–4.8)	7.6 (6.8–8.3)	17.9 (14.3–21.5)
**Gender**					
Men	23.7 (22.8–24.5)	20.2 (19.4–21.0)	13.4 (12.3–14.6)	17.1 (16.0–18.2)	35.4 (33.4–37.5)^§§^
Women	14.0 (13.3–14.7)	13.0 (12.3–13.7)	7.3 (6.5–8.0)	12.5 (11.5–13.5)	32.5 (29.9–35.2)
**Race/Ethnicity**					
White, non-Hispanic	19.6 (18.9–20.2)	17.4 (16.8–18.0)	11.4 (10.5–12.2)	14.3 (13.5–15.1)^§§^	35.1 (33.3–37.0)
Hispanic	18.5 (17.2–19.7)	13.3 (12.2–14.4)	6.0 (5.0–7.0)	16.1 (13.9–18.4)	28.6 (25.1–32.2)
Other, non-Hispanic	15.2 (12.8–17.6)	16.7 (14.4–19.1)	9.3 (6.7–11.8)	15.8 (12.3–19.4)	41.9 (33.3–50.5)
Black, non-Hispanic	12.7 (9.8–15.6)	17.9 (14.8–21.1)	10.5 (6.7–14.3)	18.3 (13.6–23.1)	44.4 (32.2–56.7)
**Education level**					
Less than high school	16.2 (14.2–18.2)	14.5 (12.6–16.4)	8.5 (6.7–10.2)	17.9 (13.6–22.2)	31.4 (24.8–37.9)
High school or GED	19.1 (17.9–20.2)	19.7 (18.5–20.1)	12.3 (11.0–13.7)	20.1 (18.0–22.1)	37.5 (34.1–40.9)
Some post-high school	19.3 (18.3–20.4)	18.4 (17.4–19.4)	10.8 (9.6–12.0)	17.0 (15.6–18.5)	36.8 (33.8–39.9)
College graduate	18.8 (18.0–19.6)	13.4 (12.7–14.1)	6.5 (5.5–7.4)	10.8 (10.0–11.6)	30.7 (28.4–33.0)

**Abbreviations:** CI = confidence interval; GED = general educational development certificate.

* Age-standardized to the 2000 U.S. population unless otherwise noted.

^†^ Current marijuana use is defined as any marijuana use, including hashish, reported on at least 1 day during the past 30 days.

^§^ Drinking patterns were categorized into three groups: 1) binge drinking (consumed four or more drinks for women or five or more drinks for men, on an occasion, one or more times in the past 30 days); 2) current non–binge drinking (consumed one or more drinks of any alcoholic beverage ≥1 day in the past 30 days but did not report any binge drinking); and 3) nondrinking (did not consume any alcoholic beverages in the past 30 days).

^¶^ All categorical differences are significant (p<0.05) unless otherwise noted.

** Weighted to the Colorado population of noninstitutionalized adults.

^††^ Crude and age-specific percentages are not age-standardized.

^§§^ Marijuana use among persons who binge drank did not vary significantly by gender. Marijuana use among non–binge drinkers did not vary significantly by race/ethnicity.

Independent of drinking pattern, cigarette smokers were more likely than nonsmokers were to use marijuana (Figure). The prevalence of marijuana use among persons who binge drank and smoked cigarettes (48.1%) was twice that of nondrinkers who smoked cigarettes (24.4%); however, among persons who did not smoke cigarettes, the prevalence of marijuana use was approximately four times as high among persons who binge drank (29.7%) as among nondrinkers (7.5%).

**FIGURE:**
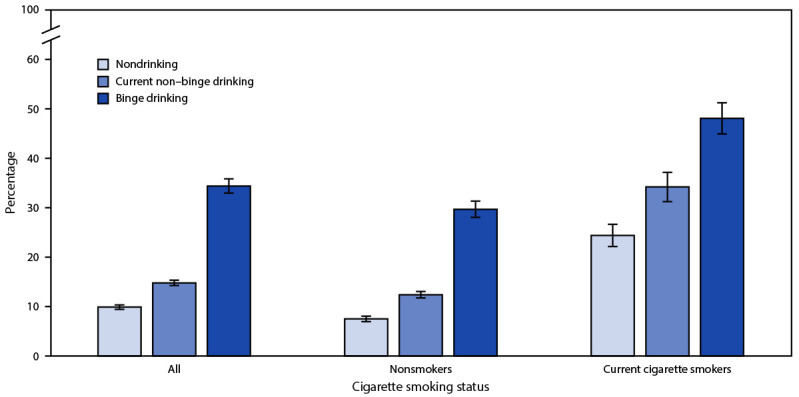
Age-standardized* prevalence^†^ of current marijuana use^§^ in the past 30 days, by drinking pattern^¶^ and cigarette smoking status** — Colorado Behavioral Risk Factor Surveillance System, 2015–2019 * Age-standardized to the 2000 U.S. population. ^†^ With 95% confidence intervals indicated by error bars. ^§^ Current marijuana use is defined as any marijuana use, including hashish, reported on ≥1 day during the past 30 days. ^¶^ Drinking patterns were categorized into three groups: 1) binge drinking (consumed four or more drinks for women or five or more drinks for men, on an occasion, one or more times during the past 30 days); 2) current non–binge drinking (consumed one or more drinks of any alcoholic beverage ≥1 day during the past 30 days but did not report any binge drinking); and 3) nondrinking (did not consume any alcoholic beverages during the past 30 days). ** Current cigarette smoking is defined as having smoked at least 100 cigarettes in lifetime and currently smoking cigarettes on some days or every day.

## Discussion

During 2015–2019, one third of adults in Colorado who reported binge drinking also reported using marijuana, consistent with other studies that have shown that persons who binge drank are more likely to use other substances, including marijuana, than are nondrinkers (*4*). The study findings indicate that persons who binge drank were more likely to report marijuana use than were nondrinkers, and the magnitude of this relationship varied by cigarette smoking status. Independent of drinking pattern, persons who smoked cigarettes were more likely to report marijuana use than were nonsmokers. The higher prevalence of marijuana use among persons who reported binge drinking, were younger, and who smoked cigarettes also aligns with other research findings (*5**,**6*).

In this study, approximately one half of adults who binge drank and currently used marijuana reported daily or near daily marijuana use, which is similar to the prevalence of daily or near daily marijuana use among all Colorado adults who use marijuana.*** Although this study did not specifically assess respondents’ use of alcohol and marijuana on the same occasion, another study of U.S. adults found that the prevalence of using both substances on the same occasion was twice as high as the prevalence of using both substances, though not on the same occasion (*7*). A 2017 National Academies of Sciences report found mixed evidence regarding whether using alcohol and marijuana on the same occasion increased risk of harms such as motor vehicle crashes, suggesting a need for more research (*8*). A literature review by CDPHE also documented the mixed evidence; however, the majority of the 10 studies reviewed (including two of higher quality) found an association between using alcohol and marijuana on the same occasion and increased impairment and motor vehicle crash risk (*9*).

The findings in this report are subject to at least three limitations. First, self-reported data on substance use might be underreported because of recall and social desirability biases; therefore, the estimates presented might be conservative. Second, there might be nonresponse or selection biases in the characteristics of persons who choose to participate in the BRFSS survey. Finally, these findings cannot be generalized to the United States because they represent adults in one state only in which nonmedical adult marijuana use is legal. The association between alcohol and marijuana use likely differs across jurisdictions because of local norms, laws, and policies.

This is the first study to assess the relationship between binge drinking and marijuana use among a representative sample of adults in Colorado. Excessive alcohol use and nonmedical marijuana use can be associated with negative health outcomes (*8*,*10*). Because of the evolution of nonmedical marijuana legalization across the United States and limited evidence on the short-term and long-term chronic effects of using alcohol and marijuana on the same occasion (*8*), continued surveillance across the lifespan of excessive alcohol use and marijuana use among persons who drink might be important to guide states in the prevention of alcohol-related harms. Adding questions to state surveillance systems on the use of alcohol, marijuana, and other substances (e.g., opioids) on the same occasion could strengthen the surveillance for risk factors or health risks associated with using multiple substances during a single occasion. To reduce excessive alcohol and tobacco use and reduce alcohol- and tobacco-related harms, the Community Preventive Services Task Force^†††^ recommends the use of evidence-based strategies such as increasing prices and reducing access. Similar strategies of limiting availability and increasing prices of marijuana (in states where marijuana sale and use is legal) might also be effective in reducing marijuana use and its potential harms.

**Summary**

**What is already known about this topic?**

Retail (nonmedical) marijuana sales began in Colorado on January 1, 2014. Adults who binge drink are more likely to use other substances than are nondrinkers.

**What is added by this report?**

During 2015–2019, one third (34.4%) of Colorado adults who binge drank used marijuana compared with one tenth (9.9%) of nondrinkers.

**What are the implications for public health practice?**

Adding questions to state surveillance systems on alcohol, marijuana, and other substance use on the same occasion could strengthen the surveillance for risk factors or risks associated with using multiple substances. The Community Preventive Services Task Force recommends evidence-based strategies (e.g., increasing prices or reducing access) to reduce excessive drinking, tobacco use, and related harms. Similar strategies might also be effective for reducing marijuana use and its potential harms.

## Footnotes

* Excessive drinking is defined as binge drinking (four or more drinks for women or five or more drinks for men on an occasion), high weekly consumption (eight or more drinks for women or ≥15 drinks for men in a week), and any drinking by pregnant women or persons aged <21 years. https://www.cdc.gov/alcohol/fact-sheets/alcohol-use.htm

†
https://www.cdc.gov/brfss/data_documentation/index.htm

§ Determined by responses to the question, “During the past 30 days, on how many days did you use marijuana or hashish?”

¶ Defined as having four or more drinks for women or five or more drinks for men, on an occasion, one or more times in the past 30 days, determined by responses to the core question, “Considering all types of alcoholic beverages, how many times during the past 30 days did you have X (X = 5 for men, X = 4 for women) or more drinks on an occasion?” One drink is equivalent to a 12-ounce beer, a 5-ounce glass of wine, or a drink with one shot of liquor.

** Defined as having one or more drinks of any alcoholic beverage ≥1 day in the past 30 days but not reporting binge drinking, determined by responses to the core questions, “During the past 30 days, how many days per week or per month did you have at least one drink of any alcoholic beverage such as beer, wine, a malt beverage or liquor?” and “Considering all types of alcoholic beverages, how many times during the past 30 days did you have X (X = 5 for men; X = 4 for women) or more drinks on an occasion?”

†† Defined as no reported alcohol use in the past 30 days, determined by response to the core question, “During the past 30 days, how many days per week or per month did you have at least one drink of any alcoholic beverage such as beer, wine, a malt beverage or liquor?”

§§ Current cigarette smoking is defined as adults who have smoked at least 100 cigarettes in their entire life and now smoke cigarettes on some days or every day and determined by responses to the core questions, “Have you smoked at least 100 cigarettes in your entire life?” and “Do you now smoke cigarettes every day, some days, or not at all?” Adults who did not meet these criteria were categorized as cigarette nonsmokers.

††† https://www.thecommunityguide.org/topic/excessive-alcohol-consumption; https://www.thecommunityguide.org/topic/tobacco
